# ADFP promotes cell proliferation in lung adenocarcinoma via Akt phosphorylation

**DOI:** 10.1111/jcmm.16136

**Published:** 2020-11-29

**Authors:** Xia Meng, Ruiying Sun, Wei Wang, Na Zhang, Shiguang Cao, Boxuan Liu, Ping Fang, Shanshan Deng, Shuanying Yang

**Affiliations:** ^1^ Department of Respiratory and Critical Care Medicine, The Second Affiliated Hospital Xi'an Jiaotong University Xi'an, Shaanxi China; ^2^ Department of Pathology, The Second Affiliated Hospital Xi'an JiaoTong University Xi'an, Shaanxi China; ^3^ Department of Nuclear Medicine, The Second Affiliated Hospital Xi'an Jiaotong University Xi'an, Shaanxi China

**Keywords:** ADFP, lung adenocarcinoma, phosphorylation of Akt, proliferation

## Abstract

Previously, we identified differentially expressed proteins, including ADFP, between lung adenocarcinoma (LAC) tissue and paired normal bronchioloalveolar epithelium. In this study, we investigated the role of ADFP in LAC. ADFP levels in the serum of patients with lung cancer and benign diseases were measured by enzyme‐linked immunosorbent assays (ELISA). shRNA was used to knock‐down or overexpress ADFP in A549 and NCI‐H1299 cells. The biological function of ADFP and its underlying mechanisms was evaluated in vivo and in vitro. ADFP was highly expressed in the serum of lung cancer patients, especially those with LAC. ADFP promoted cell proliferation and up‐regulated the p‐Akt/Akt ratio in A549 and NCI‐H1299 cells in vitro. Furthermore, in nude mice, ADFP promoted tumour formation with high levels of p‐Akt/Akt, Ki67 and proliferating cell nuclear antigen (PCNA). Similar to the effect of ADFP knock‐down, MK‐2206 (a phosphorylation inhibitor of Akt) reduced A549 and NCI‐H1299 cell proliferation. In ADFP‐overexpressing A549 and NCI‐H1299 cells, proliferation was suppressed by MK‐2206 and returned to the control level. ADFP did not regulate invasion, migration or adhesion in LAC cells. Together, these results suggest that ADFP promotes LAC cell proliferation in vitro and in vivo by increasing Akt phosphorylation level.

## 1 INTRODUCTION

Lung cancer is the most frequent malignant tumour with the highest morbidity and mortality rates worldwide. The analysis of GLOBOCAN 2018 data^1^ showed that 2.09 million people would be diagnosed with lung cancer in 2018, accounting for 11.6% of the total cancer cases, and the number of deaths would increase to 1.76 million (18.4% of total). Lung cancer causes significant social and economic burdens and has become a serious global public health concern.^2^ Lung adenocarcinoma (LAC) is the most common pathological type, which is characterized by many clear and prominent driver mutations, such as TP53, KRAS, EGFR, NF1, BRAF and MET. Meanwhile, oncogenic fusions and in‐frame rearrangements with several kinases were reported in LAC, including ALK, ROS1 and RET. Somatic copy number alterations are fairly prevalent, occurring both as early and late events during tumour evolution, including amplifications in EGFR, MET, KRAS, ERBB2 and MDM2, and deletions in LRP1B, PTPRD and CDKN2A. Detection of these genomic alterations has substantially changed the setting of lung cancer diagnosis and treatment.

The transformation from normal tissues to malignant tumours is strictly regulated by the type, location and quantity of proteins that perform biological functions. Comparative proteomics is a powerful method for identifying a range of proteins that are differentially expressed between normal and tumour tissues. We previously purified LAC cells and normal bronchial alveolar epithelial cells from surgical LAC tissues and matched normal lung tissues, respectively, by laser‐capture microdissection. The isobaric tags for relative and absolute quantification (iTRAQ) and two‐dimensional liquid chromatography‐tandem mass spectrometry (2D‐LC‐MS/MS) were used to screen the differentially expressed membrane proteins. A total of 568 proteins were identified, including ADFP (adipophilin, ADRP, PLIN2), which was up‐regulated by 2.46‐fold in LAC.^3^ Further, we measured ADFP levels in 62 cases of primary lung cancer tissues and 24 cases of normal lung tissue by immunohistochemical (IHC) staining and found that ADFP was significantly highly expressed in LAC.^4^

ADFP is called adipose differentiation‐related protein and has a molecular weight of 50 kDa. The human ADFP gene is located in 9 p22.1 encoding 437 amino acids. ADFP is a membrane‐associated protein belonging to the ancient PAT protein family and was originally considered to be a unique protein on the surface of fat droplets in fat cells.^5^ Since then, ADFP has been detected in most tissue cells, such as breast, macrophages, liver, heart, intestinal mucosa, skeletal muscle, pancreas and others. ADFP is also involved in cellular fatty acid uptake, lipid droplet formation and lipid storage. Several conserved regions in the protein structure have the potential to bind lipids.^6^, ^7^, ^8^, ^9^, ^10^, ^11^, ^12^ There is a dynamic balance between the decomposition and storage of fat in lipid droplets. Perilipin 5 (PLIN5) distributed on the surface of lipid droplets allows triglyceride lipase and hormone‐sensitive lipase to enter the lipid droplets, promoting lipid degradation and free fatty acid consumption. ADFP hinders the entry of lipase into lipid droplets, resulting in the accumulation of lipids in lipid droplets. PLIN5 and ADFP help to maintain equilibrium on the surface of lipid droplets.^13^, ^14^ In the absence of lipids to bind, ADFP is easily degraded by the proteasome; thus, ADFP is positively correlated with intracellular lipid content. Therefore, ADFP is used as an index for evaluating lipid accumulation under physiological and pathological conditions. Abnormal ADFP function and expression are also associated with a variety of diseases related to disordered lipid metabolism.^14^, ^15^

Recent studies showed that ADFP is highly expressed in Burkitt lymphoma,^16^ colorectal cancer,^17^ liver cancer,^18^ skin cancer,^19^ malignant melanoma and clear cell renal cell carcinoma.^20^, ^21^ Therefore, ADFP may play a role as an oncogene during tumorigenesis and development. However, high expression of ADFP in urine and kidney tissues is associated with a good prognosis of patients with clear cell renal cell carcinoma.^20^, ^22^, ^23^ Bioplasmonic paper‐based assays that can quickly detect the content of ADFP in the urine of patients are expected to be widely used in clinical practice.^24^ Cao et al knocked down ADFP in the human kidney cancer cell line A498 using an oligonucleotide sequence and observed enhanced proliferation, invasion and migration abilities.^23^ Therefore, ADFP may also play a role as a tumour suppressor gene. Significantly, the role of ADFP in LAC remains unclear. In our previous research, the sample number of early‐stage lung cancer patients was insufficient. Limitations of it included the heterogeneity of the population used and the small sample size. Thus, ADFP level in the peripheral blood of lung cancer patients, especially LAC patients, is still to be observed and more samples are needed to be included in the study.

## 2 MATERIALS AND METHODS

### 2.1 Clinical samples and enzyme‐linked immunosorbent assay (ELISA)

In this study, 177 lung cancer patients (79 cases, LAC; 55 cases, lung squamous carcinoma; and 43 cases, small‐cell lung cancer) and 22 patients with benign lung diseases (8 cases, pneumonia; 5 cases, tuberculosis; 2 cases, lung abscess; 3 cases, chronic obstructive pulmonary disease; 2 cases, pulmonary fibrosis; 1 case, bronchiectasis; and 1 case, asthma) were selected from the Department of Respiratory and Critical Care Medicine in The Second Affiliated Hospital of Xi'an Jiaotong University (Xi'an, China) between 2014 and 2017. The diagnoses of lung cancer were based on clinical and histological examinations. The Clinical Research Ethics Committee of the Second Affiliated Hospital of Xi'an Jiaotong University approved the study, and every patient provided written informed consent. Peripheral blood (3 mL) from each patient was collected and centrifuged. The serum was separated, stored at − 80°C and used for ELISA. The concentrations of ADFP protein in serum samples were determined by ELISA using an ADFP ELISA Kit (Elabscience Biotechnology) according to the manufacturer's recommendations and measured by a Microplate Absorbance Reader (Thermo).

### 2.2 Cells culture

Human LAC cell lines A549 and NCI‐H1299 were obtained from the Cell Bank of the Chinese Academy of Science (China). Both cell lines were cultured in RPMI1640 (Gibco) medium with 10% foetal bovine serum (Biological Industries, Israel) and maintained in a cell incubator (Thermo) at 5% CO_2_ and 37°C.

### 2.3 Lentiviral transduction

The pLV[shRNA]‐EGFP:T2A:Puro‐U6 knock‐down lentiviral vector for ADFP, pLV[Exp]‐EGFP:T2A:Puro‐EF1A overexpressing lentiviral vector for ADFP and respective control lentiviral vectors were obtained from Cyagen Biosciences. The efficiency of transduction was analysed by GFP fluorescence.

### 2.4 MK‐2206

MK‐2206, a phosphorylation inhibitor of Akt (Akt1, Akt2 and Akt3), was obtained from Selleck Chemicals. MK‐2206 powders were dissolved by dimethyl sulfoxide (DMSO) and diluted to 1 mM (5% DMSO) by RPMI1640. The solution was stored at − 80°C. Where indicated, cells were exposed to MK‐2206 after being cultured for 24 h, which was recorded as the start of the experiment.

### 2.5 Real‐time quantitative polymerase chain reaction (RT‐qPCR)

Total RNA was extracted using an RNA Extraction Kit (Takara). The PrimeScript™RT Master Mix Kit (Takara) was used for RNA reverse transcription. The primer sequences were designed and synthesized (Sangon Biotech, China) as follows: GAPDH (forward 5'‐GTCTCCTCTGACTTCAACAGCG‐3', reverse 5'‐ACCACCCTGTTGCTGTAGCCAA‐3') and ADFP (forward 5'‐ GATGGCAGAGAACGGTGTGAAG‐3', reverse 5'‐AGGCATAGGTATTGGCAACTGC‐3'). RT‐qPCR analysis was performed with the TB Green^®^ Premix Ex Taq™ II Kit (Takara, Japan). The 2^‐ΔΔCt^ method was used to determine the relative quantitative gene expression levels of ADFP, and GAPDH was used as a reference.

### 2.6 Western blotting

Total protein was extracted using RIPA buffer (Beyotime) containing a protease inhibitor (Roche). The proteins were separated by SDS‐PAGE and transferred onto PVDF membranes (Millipore). The membranes were blocked with 10% milk for 2‐4 h and incubated with primary antibodies at 4°C overnight. The primary antibodies used were as follows: anti‐ADFP (1:500; AbGene, China), anti‐p‐Akt (Ser 473) (1:1000; CST), anti‐Akt (1:1000; CST), anti‐p‐STAT3 (1:1000; Abcam), anti‐STAT3 (1:1000; Abcam), anti‐p‐ERK (1:1000; CST, anti‐ERK (1:1000; CST), anti‐β‐catenin (1:1500; Santa, anti‐NFκB (1:1500; Abcam), anti‐Bcl‐2 (1:1000; Abcam), anti‐Bax (1:1500; Abcam) and anti‐GAPDH (1:2000; Abcam). After washing in TBST, the membranes were incubated with an anti‐rabbit IgG secondary antibody (1:10 000; Solarbio) at room temperature for 2 h. Lastly, signals were detected using an HRP Chemiluminescent Kit (Millipore) and CCD camera image system (Bio‐Rad).

### 2.7 CCK‐8 assay

Cells were seeded in a 96‐well plate (Corning) at a density of 6 × 10^3^ cells/100 μL complete medium per well and cultured for 24‐96 h. Next, 10 μL of the CCK‐8 reagent was added to each well and incubated for 1 h at 37°C. The optical density was measured using an absorbance microplate reader (Thermo, USA) at a wavelength of 450 nm.

### 2.8 5‐Ethynyl‐2'‐deoxyuridine (EdU)

Cells were seeded in a 96‐well plate (Corning) at a density of 3 × 10^3^ cells/100 μL complete medium per well and cultured for 24 h, followed by serum starvation in serum‐free medium for 24 h. Then, cells were labelled with EdU and cultured for 24 h. Apollo staining and DNA staining were performed according to the protocol described by the manufacturer of the EdU kit (Ribobio). Finally, cells were observed under a fluorescence microscope (Leica). The Hoechst33342 nuclear staining signal was excited by blue light, representing the viable cells containing DNA (total cells), and the Apollo staining signal was excited by red light, representing the cells with replicated DNA (proliferating cells).

### 2.9 Plate colony formation

Cells were seeded in a 6‐well plate (Corning) at a density of 200 cells with 3 mL complete medium per well and cultured for 10 days. Then, cells were fixed in methanol and stained with 0.1% crystal violet, and the number of colonies was counted.

### 2.10 Flow cytometry

Cells were seeded in a 6‐well plate (Corning) at a density of 1 × 10^5^ cells with 3 mL complete medium per well and cultured for 24 h, followed by serum starvation in serum‐free medium for 24 h. Then, cells were cultured in complete medium for 24 h and processed according to the protocol described by the manufacturer of the PI Cell Cycle Analysis Kit (7sea Biotech). Samples were analysed by a flow cytometer (BD).

### 2.11 Transwell migration and invasion assays

Cells were seeded in a 24‐well transwell insert (Millipore) that was either uncoated (migration assay) or coated (invasion assay) with Matrigel at a density of 1.5 × 10^5^ cells (migration assay) or 5 × 10^5^ cells (invasion assay) with 200 μL serum‐free medium per transwell insert. Complete medium (800 μL) was added into each lower chamber of the 24‐well plate (Corning) as an attractant. Cells were incubated for 24‐48 h. Afterwards, cells in the upper chamber were removed, and cells that had migrated/invaded through the membrane were fixed in methanol and stained with 0.1% crystal violet. The number of migrated/invaded cells was counted under a microscope (Nikon, Japan).

### 2.12 Wound healing assay

Cells were seeded in a 6‐well plate at a density of 1 × 10^5^ cells with 3 mL complete medium per well and cultured until confluent. Then, cells were scratched using a 100 μL tip and cultured in serum‐free medium for 24‐72 h. The wound area was calculated and analysed under a microscope (Nikon, Japan).

### 2.13 Adhesion assay

Cells were seeded in a 96‐well plate (Corning) coated with Matrigel at a density of 5 × 10^4^ cells with 200 μL complete medium per well and incubated for 1 h. Afterwards, cells were fixed in methanol and stained with 0.1% crystal violet. The number of adhered cells was counted under a microscope (Nikon, Japan).

### 2.14 Tumour formation in nude mice

Female athymic nude mice (BALB/c, 3‐4 weeks old) were purchased from and fed in the Xi'an Jiaotong University Medical Laboratory Animal Center. All experiments were approved by Xi'an Jiaotong University. The mice were divided into groups of five. Then, 5 × 10^7^ cells were injected into the subcutis under the right armpit of each mouse. The long and short diameters of tumours were measured every 3 days after tumour formation. After 1 month, the tumours were observed using an Animal Vivo Imaging Machine (Perkin Elmer). Then, mice were killed, and the tumours were weighed and fixed in formalin for IHC staining.

### 2.15 IHC staining

Tissues were dehydrated in a graded series of alcohol (50%, 70%, 85%, 95% and 100%) and dimethylbenzene, followed by paraffin embedding at 65°C. Blocks of tissues were cut into slices and dried on glass slides at 60°C for 12 h. Then, the slides were dewaxed with dimethylbenzene and gradient alcohol. Antigen retrieval was performed by microwaving sections in citrate buffer for 2 min. Slides were incubated with goat serum for 30 min at 37°C. Primary antibodies against p‐Akt (Ser 473) (1:50; CST), Akt (1:50; CST), Ki67 (1:50; Abcam USA) and proliferating cell nuclear antigen (PCNA) (1:50; CST) were incubated with slides at 4°C overnight. The secondary anti‐IgG antibody was added and incubated at 37°C for 30 min. The slides were stained with 3,3'‐diaminobenzidine and haematoxylin and then washed with flowing water. Afterwards, the slides were dehydrated with gradient alcohol (70%, 80%, 90% and 100%) and dimethylbenzene. Finally, the slides were sealed with neutral balsam and coverslips.

### 2.16 Statistical analysis

SPSS Statistics 23 (SPSS Inc) was used for all statistical analyses. Wilcoxon signed‐rank, and Kruskal‐Wallis tests were used to determine differences in serum ADFP levels among cancer and control groups. Data were presented as Median (Q1, Q3). The comparisons between two groups or among three groups were performed using a t test or ANOVA. The differences in the CCK‐8 assay and tumour volume analyses were determined by repeated measures ANOVA. Data were presented as the mean ± SD. All statistical tests were two‐sided, and values of *P* < .05 were considered significantly different.

## 3 RESULTS

### 3.1 ADFP is up‐regulated in the peripheral blood serum of patients with LAC

We first collected peripheral venous blood samples from 177 patients with lung cancer (including 79 LAC, 55 lung squamous carcinoma and 43 small‐cell lung cancer) and 22 patients with benign lung diseases. The ADFP protein levels in serum were detected by ELISA. The results showed that the ADFP level was 91.84 ng/mL (77.38 ng/mL, 107.74 ng/mL) in the peripheral blood serum of patients with lung cancer and 80.63 ng/mL (48.44 ng/mL, 96.18 ng/mL) in patients with benign diseases. The difference was statistically significant (*P* < .05). Compared with the benign disease group, the ADFP levels in the LAC and small‐cell lung cancer groups were significantly higher (*P* < .05). There was no statistical difference between the lung squamous cell carcinoma group and the benign disease group (Table 1). We further compared ADFP expression among the three common pathological types of lung cancer. The ADFP level in small‐cell lung cancer was higher than in LAC (Bonferroni corrected *P* < .05) and squamous cell carcinoma (Bonferroni corrected *P* < .05). There was no difference between LAC and lung squamous cell carcinoma.

**Table 1:** Serum ADFP levels in different pathological types of lung cancer [Median (Q1, Q3)]

Groups	*N*	ADFP (ng/mL)	*P* value
Benign lung diseases	22	80.63 (48.44, 96.18)	—
Lung cancer	177	91.84 (77.38, 107.74)	.008^a^
LAC	79	89.22 (74.65, 106.35)	.037^a^
Lung squamous carcinoma	55	85.64 (75.28, 104.86)	.069^a^
Small‐cell lung cancer	43	99.04 (89.88, 111.84)	.001^a^

^a^ Compared with benign lung diseases group. Wilcoxon signed‐rank test.

The relationship between serum ADFP levels and the clinical characteristics of patients with LAC was analysed. Patients with regional lymph node invasion had higher ADFP serum levels than those without (*P* < .05). There was also no significant difference among patients with different distant metastasis status, genders, ages, smoking indexes and clinical stages (Table 2).

**Table 2:** LAC patients’ serum ADFP level and clinical characteristic [Median (Q1, Q3)]

Parameter	*N*	ADFP(ng/mL)	*P* value
Gender			
Male	45	89.22 (76.17, 107.28)	.759^a^
Female	34	89.81 (72.43, 106.78)	
Age			
<60	30	85.83 (71.83, 102.36)	.551 ^a^
≥60	49	90.84 (75.95, 108.29)	
Smocking index			
0	44	88.60 (70.01, 102.45)	.382^b^
1‐400	8	87.70 (76.59, 117.27)	
400‐800	14	98.40 (75.50, 116.67)	
800‐1200	11	98.27 (82.72, 126.95)	
>1200	2	—	
Regional lymph node invasion			
Negative	31	79.21 (68.58, 97.98)	.020 ^a^
Positive	48	95.37 (80.09, 108.40)	
Distant metastasis			
Negative	20	79.41 (67.18, 102.97)	.107 ^a^
Positive	59	91.94 (76.94, 108.51)	
AJCC stage			
Ⅰ/Ⅱ	13	79.60 (59.74, 104.63)	.239 ^a^
Ⅲ/Ⅳ	66	91.34 (75.29, 108.18)	

^a^ Wilcoxon signed‐rank tests.

^b^ K‐W test.

### 3.2 ADFP promotes LAC cell proliferation

**Figure 1:**
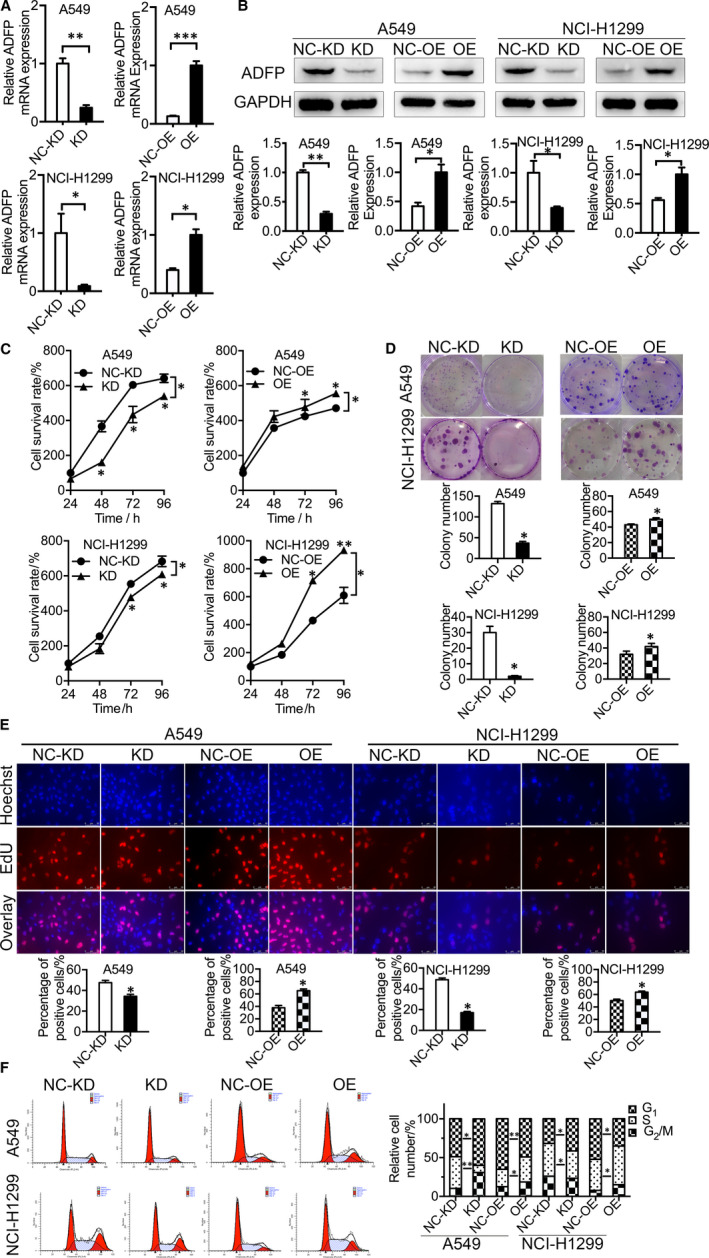
ADFP showed tumour‐promoting ability in LAC cells. A and B, A549 and NCI‐H1299 cells were stably transduced with ADFP knock‐down (KD) and ADFP overexpression (OE) lentiviral vectors. Control groups (NC‐KD and NC‐OE) were transduced with the corresponding empty vector. Overexpression and knock‐down of ADFP were confirmed by RT‐qPCR and Western blotting. C, ADFP knock‐down significantly reduced cell viabilities in the CCK‐8 assay compared with those of control A549 and NCI‐H1299 cells. ADFP overexpression significantly promoted cell viabilities in the CCK‐8 assay compared with that of the control A549 and NCI‐H1299 groups. D, ADFP knock‐down significantly decreased colony numbers compared with those of control A549 and NCI‐H1299 cells. ADFP overexpression significantly increased colony numbers compared with those of the control A549 and NCI‐H1299 groups. E, ADFP knock‐down significantly decreased the proportion of EdU‐positive cells compared with that of control A549 and NCI‐H1299 cells. ADFP overexpression significantly increased the proportions of EdU‐positive cells compared with those of the control A549 and NCI‐H1299 groups. Blue represents the cell nucleus, and red represents DNA replication. F, Cell cycle was analysed by flow cytometry. ADFP reduced the proportions of cells in the G1 phase and increased the fraction of cells in the S phase. Bars represent the SE of the mean ± SD from three independent experiments. *P < .05 and **P < .01 and ***P < .001

The up‐regulation of ADFP in the serum of clinical LAC patients suggested the potential tumour‐promoting ability of ADFP in the progression of LAC. To evaluate the role of ADFP in LAC progression, we selected A549 and NCI‐H1299 LAC cell lines for further research. We established stable ADFP knock‐down A549 cells, ADFP knock‐down NCI‐H1299 cells, ADFP‐overexpressing A549 cells, ADFP‐overexpressing NCI‐H1299 cells and respective control groups by lentivirus transfection. The efficiencies of knock‐down and overexpression were verified by fluorescence microscopy (Figure S1A), RT‐qPCR (Figure 1A) and Western blotting (Figure 1B).

ADFP overexpression in A549 and NCI‐H1299 cells significantly promoted cell growth, as determined by the CCK‐8 assay, whereas ADFP knock‐down suppressed cell growth (Figure 1C). The role of ADFP was further confirmed by the increased colony formation ability of ADFP‐overexpressing A549 and NCI‐H1299 cells and the reduced colony formation ability of ADFP knock‐down A549 and NCI‐H1299 cells (Figure 1D). The results of EdU experiments showed that the proportions of proliferating ADFP knock‐down A549 and NCI‐H1299 cells were decreased, whereas the proportions of proliferating ADFP‐overexpressing A549 and NCI‐H1299 cells were increased (Figure 1E). Furthermore, ADFP induced S phase arrest in A549 and NCI‐H1299 cells (Figure 1F). These data collectively suggested that ADFP played a tumour‐promoting role in LAC cells.

### 3.3 ADFP does not regulate the invasion, migration or adhesion of LAC cells

**Figure 2:**
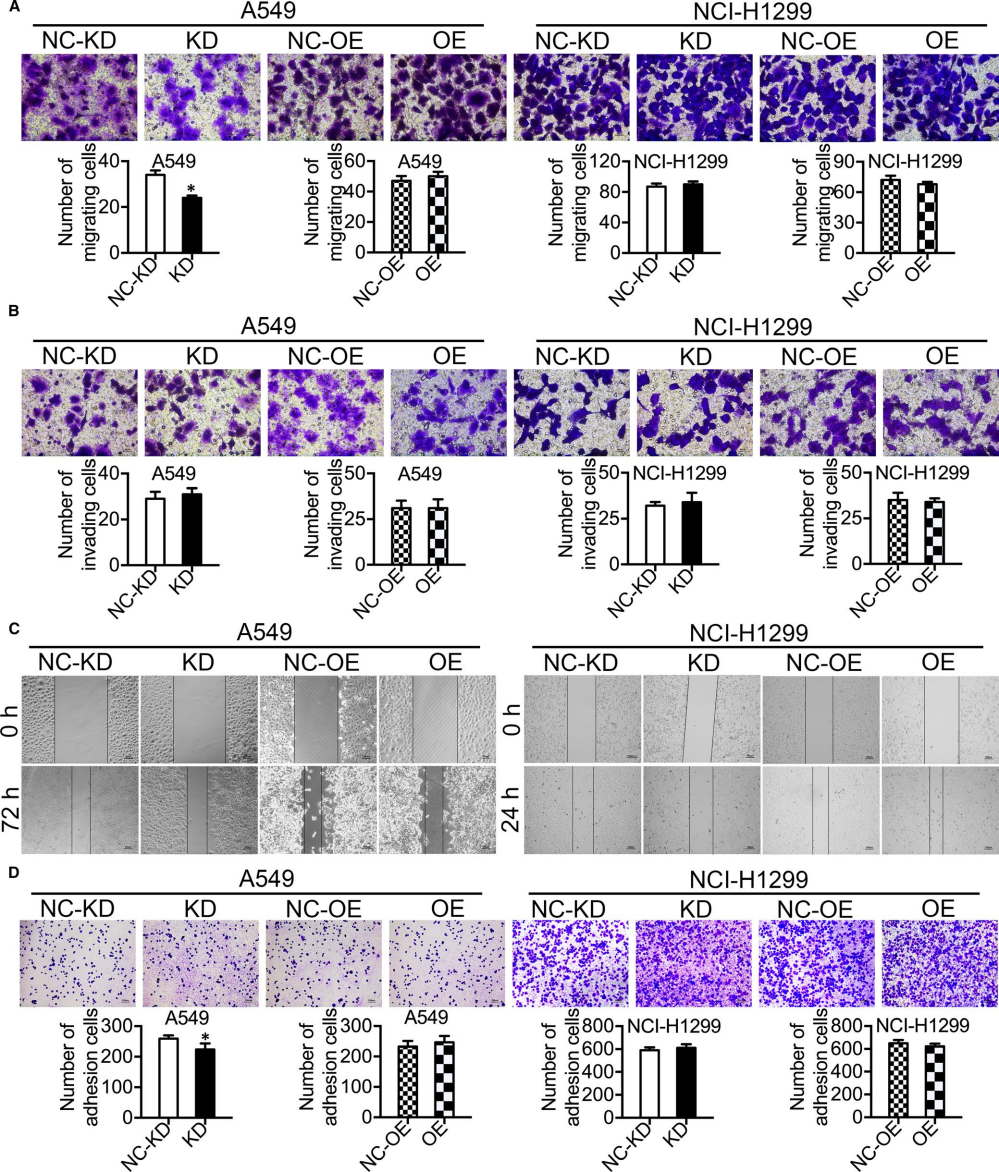
Role of ADFP in the invasion, migration and adhesion of LAC cell lines. A and B, Only ADFP knock‐down significantly reduced the cell migration ability of A549 cells, as shown by transwell studies. C, ADFP does not regulate the invasion or migration abilities of LAC cell lines, as shown by wound healing assays. D, ADFP knock‐down reduced the cell adhesion of A549 cells in adhesion assays. *P < .05

The roles of ADFP in invasion, migration and adhesion in LAC were further evaluated. In transwell assays, the migration of ADFP knock‐down A549 cells was significantly suppressed. However, ADFP knock‐down had no effect on the invasion of A549 cells, and ADFP overexpression did not affect the migration or invasion of A549 cells (Figure 2A,B). Similarly, there was no change in the migration or invasion of NCI‐H1299 cells (Figure 2A,B). In addition, the results of wound healing assays showed no significant differences between any of the groups (Figure 2C). The number of adherent ADFP knock‐down A549 cells was decreased, but there was no significant change among other groups in adhesion assays (Figure 2D).

### 3.4 ADFP promotes tumour growth in vivo

**Figure 3:**
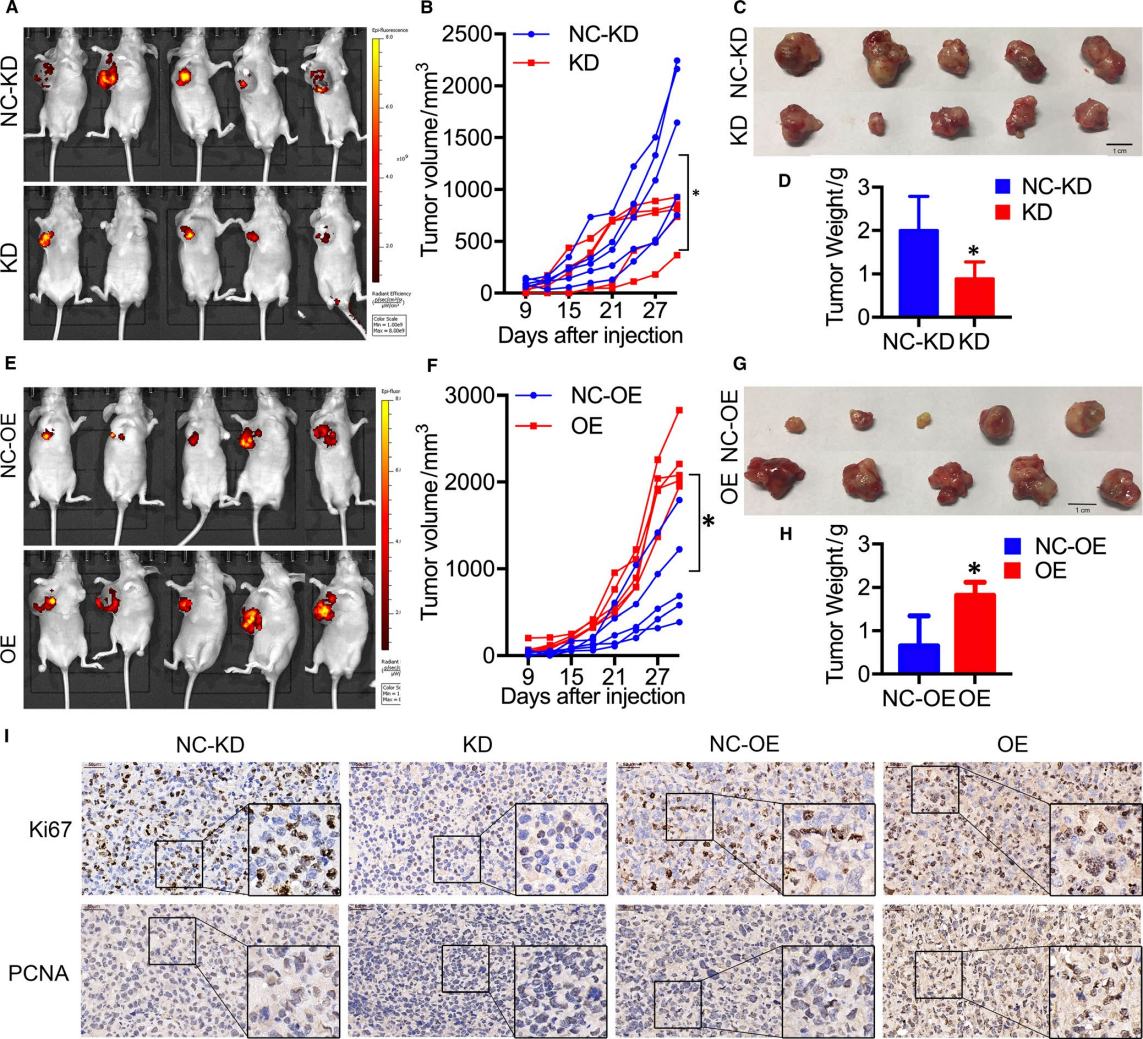
ADFP promoted tumour growth in vivo. A, Animal Vivo images of ADFP knock‐down NCI‐H1299 xenograft nude mice (GFP fluorescent image) show that the ADFP knock‐down reduced the growth of NCI‐H1299 xenografts (n = 5). B, The histogram of tumour volumes shows that ADFP knock‐down NCI‐H1299 cells formed smaller xenografts compared with those of the control groups. C, Images of NCI‐H1299 xenograft tumours show that the ADFP knock‐down impaired the growth of NCI‐H1299 xenografts in nude mice (n = 5). D, The histogram of tumour weight shows that ADFP knock‐down NCI‐H1299 cells formed smaller xenografts. E, Animal Vivo images of ADFP‐overexpressing NCI‐H1299 xenograft nude mice (GFP fluorescent image) show that the ADFP overexpression promoted the growth of NCI‐H1299 xenografts (n = 5). F, The histogram of tumour volumes shows that ADFP‐overexpressing NCI‐H1299 cells formed larger xenografts. G, Images of NCI‐H1299 xenograft tumours show that the ADFP overexpression accelerated the growth of NCI‐H1299 xenografts in nude mice (n = 5). H, The histogram of tumour weight shows that ADFP‐overexpressing NCI‐H1299 cells formed larger xenografts. I, Representative images of the IHC staining of Ki67 and PCNA in the tumours from mice injected with ADFP knock‐down and ADFP‐overexpressing NCI‐H1299 cells (40×). Bars represent the standard error of the mean ± SD. *P < .05

The role of ADFP in promoting LAC cell proliferation was verified in vitro. To explore the tumorigenic function of ADFP in vivo, we inoculated ADFP knock‐down NCI‐H1299, ADFP‐overexpressing NCI‐H1299 and their respective control cells into the hypoderm of nude mice, to establish tumour‐bearing mouse models (Figure 3A,E). Compared with control groups, the tumour volumes in the ADFP knock‐down NCI‐H1299 group were significantly smaller (Figure 3B), whereas those in the ADFP‐overexpressing NCI‐H1299 group were larger (Figure 3F). At the end of the experiment, the tumour weights of nude mice injected with ADFP knock‐down NCI‐H1299 cells were significantly decreased (Figure 3C,D), and those from mice injected with ADFP‐overexpressing NCI‐H1299 cells were significantly increased (Figure 3G,H). Ki67 and PCNA expression levels were lower in mouse tumour tissues in the ADFP knock‐down NCI‐H1299 group and higher in those of the ADFP‐overexpressing NCI‐H1299 group (Figure 3I). These data indicated that ADFP promoted LAC cell tumour proliferation in vivo.

### 3.5 ADFP functions by enhancing the Akt phosphorylation levels in LAC

**Figure 4:**
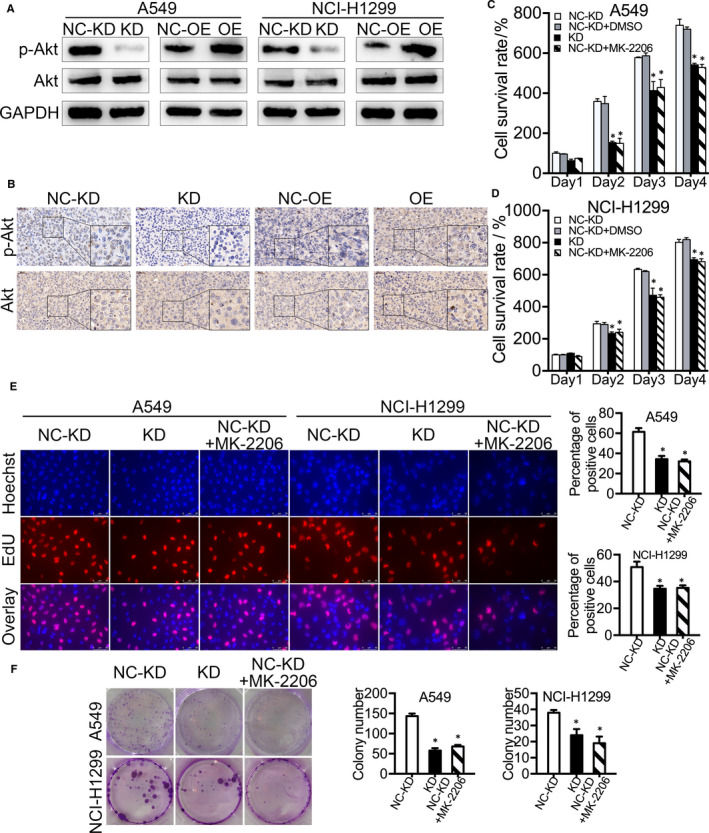
Effect of MK‐2206 on LAC cell proliferation was consistent with ADFP knock‐down. A, Expression levels of p‐Akt and Akt in ADFP knock‐down and ADFP‐overexpressing LAC cells were detected by Western blotting. B, Levels of p‐Akt and Akt in the tumours from mice injected with ADFP knock‐down and ADFP‐overexpressing NCI‐H1299 cells were detected by IHC staining (40×). C and D, MK‐2206 significantly reduced cell viability in the CCK‐8 assay, which was consistent with the ADFP knock‐down in A549 and NCI‐H1299 cells. E, MK‐2206 significantly reduced the proportion of proliferating cells, as shown by the EdU assay, which was consistent with the ADFP knock‐down in A549 and NCI‐H1299 cells. F, MK‐2206 significantly decreased the number of colonies in the plate colony formation assay, which was consistent with the ADFP knock‐down in A549 and NCI‐H1299 cells. *P < .05

**Figure 5:**
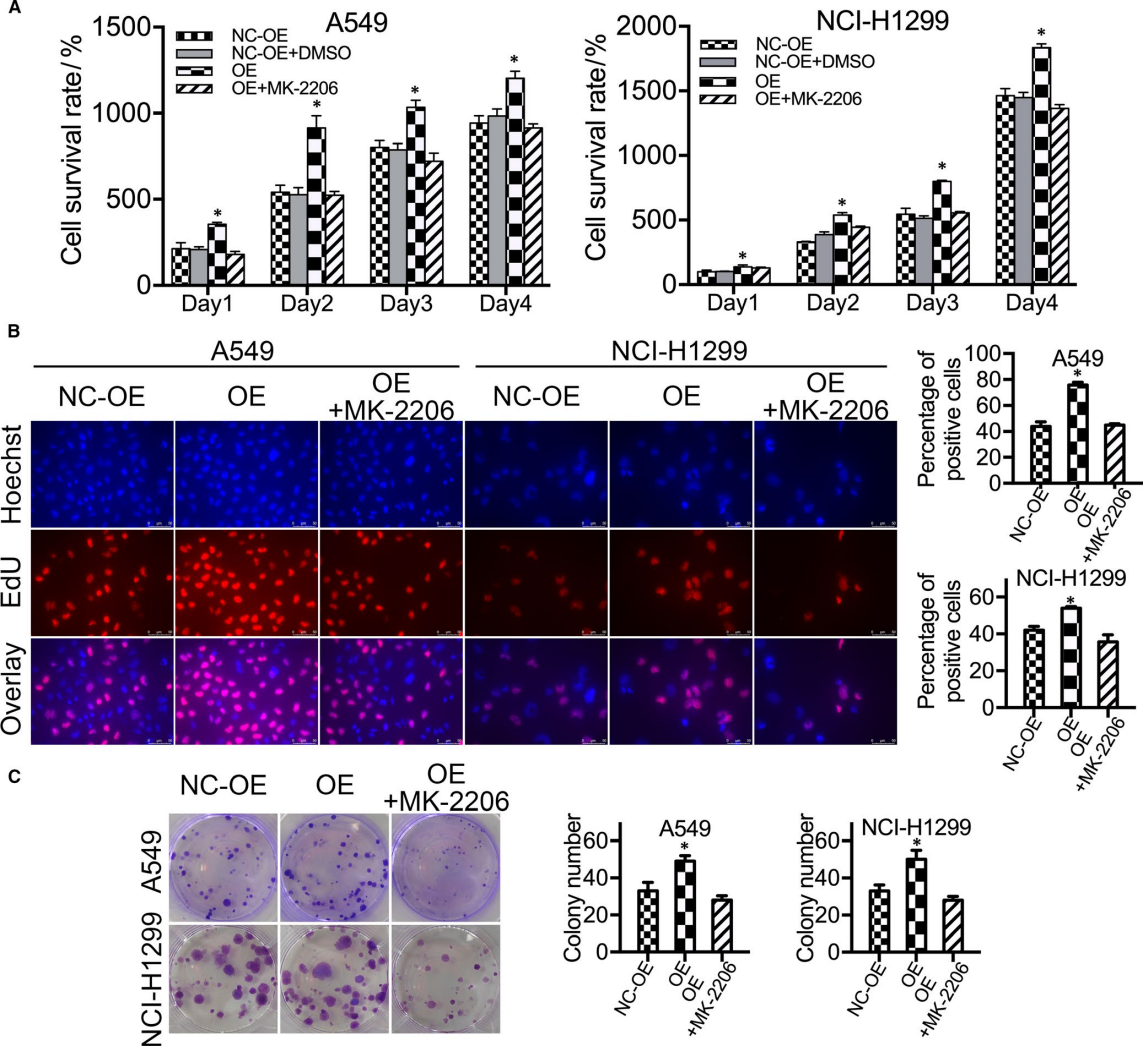
The cell proliferation ability of ADFP‐overexpressing LAC cells was suppressed by MK‐2206 back to the level of the control group. A, The cell viability of ADFP‐overexpressing LAC cells was reduced by MK‐2206 back to the level of the control group in the CCK‐8 assay. B, The proportion of proliferating ADFP‐overexpressing LAC cells was reduced by MK‐2206 back to the level of the control group, as shown by the EdU assay. C, The number of colonies formed by ADFP‐overexpressing LAC cells was reduced by MK‐2206 back to the level of the control group, as shown by the plate colony formation assay. *P < .05

To further explore the function of ADFP in LAC cell proliferation, we examined the protein expression levels of p‐Akt, Akt, p‐STAT3, STAT3, p‐ERK, ERK, β‐catenin, NF‐κB, Bcl‐2 and Bax in A549 and NCI‐H1299 cells. The expression of p‐Akt protein was decreased in ADFP knock‐down cells and increased in ADFP‐overexpressing cells (Figure 4A). There were no significant changes in Akt (Figure 4A) or other detected proteins (Figure S1B) among the different groups. In the tumour‐bearing mouse models mentioned above, p‐Akt expression levels were lower in the tumour tissues of the ADFP knock‐down NCI‐H1299 group and higher in those of the ADFP‐overexpressing NCI‐H1299 group. In contrast, Akt expression levels were similar among all groups (Figure 4B). Thus, the Akt phosphorylation level (p‐Akt/Akt) showed changes that were similar to ADFP expression. Therefore, we speculated that the function of ADFP may be related to Akt phosphorylation. To address this, we inhibited the phosphorylation of Akt in control A549 and NCI‐H1299 groups by treatment with 0.5 μM of MK‐2206 (Figure S1C). Consistent with the effect of ADFP knock‐down, MK‐2206 reduced the cell proliferation rate (Figure 4C,D), the proportion of proliferating cells (Figure 4E) and the number of colonies formed (Figure 4F). In ADFP‐overexpressing A549 and NCI‐H1299 cells, the cell proliferation rate (Figure 5A), the proportion of proliferating cells (Figure 5B) and the number of colonies formed (Figure 5C) were suppressed by MK‐2206 back to the levels of the control group.

## 4 DISCUSSION

Recent studies reported high levels of ADFP in the tissues and bodily fluids of patients with several malignant tumours.^20^, ^21^, ^25^, ^26^ ADFP was also an independent predictor of poor prognosis in patients with pancreatic ductal adenocarcinoma.^27^ In clear cell renal cell carcinoma tissues, the transcript levels and protein expression of ADFP were higher than in normal renal tissues,^25^ and its increased expression could be used as a diagnostic and prognostic factor for clear cell renal cell carcinoma. This study found significantly higher levels of ADFP in the serum of lung cancer patients, which was consistent with the results of tumour tissue analyses we previously conducted.^4^ We further analysed three common pathological types of lung cancer. Compared with patients with benign lung diseases, serum ADFP levels in patients with LAC and small‐cell lung cancer were significantly increased, but no statistical difference was found in lung squamous cell carcinoma. The ADFP level in small‐cell lung cancer was higher than in LAC. The measurement of serum ADFP levels might be helpful for the diagnosis of LAC and small‐cell lung cancer. Peripheral blood indicators have the advantages of quicker, more accessible and less trauma. However, as a membrane protein, its level is not strictly linear in peripheral blood serum and intracellular. We should be cautious in judging whether an indicator can be used for diagnosis by its peripheral blood levels. The relationship between expression at cellular level and the concentration in the serum level needs to be further explored and better experimentally demonstrated. Self‐control specimens of primary LAC tissue and peripheral blood are the most anticipated, but difficult to be obtained. In LAC, the serum ADFP levels were not statistically different among patient groups with different genders, ages and smoking indexes, which is consistent with the results in our previous study. In LAC, ADFP was associated with regional lymph node invasion. There was difference of ADFP serum level between patients with or without lymph node metastases but no difference between patients with or without distal metastases. There is evidence that lymph node and distant metastases develop through fundamentally different evolutionary mechanisms. Lymph node metastasis and distant metastasis are not completely synchronized.^28^ We consider that both of the arguments above need to be taken one stage further, for example, larger sample size, more detailed classification of clinicopathological characteristics and smaller population heterogeneity.

Previous studies found that the high ADFP level in urine and renal tissues was associated with a good prognosis in clear cell renal cell carcinoma patients,^20^, ^22^, ^23^ and the expression of ADFP in tumour tissues with a low clinical stage was significantly higher than that in tumours with a high clinical stage.^20^ Furthermore, Cao et al found that ADFP knock‐down in A498 cells (a human kidney cancer cell line) enhanced cell proliferation, migration and invasion.^23^ These results indicated that ADFP may also function as an anti‐oncogene. We explored the roles of ADFP in LAC using CCK‐8, EdU, plate cloning assays, transwell assays and adhesion assays and found that ADFP overexpression significantly increased A549 and NCI‐H1299 cell proliferation. We established tumour‐bearing mouse models with ADFP knock‐down and ADFP‐overexpressing NCI‐H1299 cells and their respective control cells. The protein levels of Ki67 and PCNA, which are specific markers of cell proliferation, were reduced in the ADFP knock‐down cells and increased in ADFP‐overexpressing cells.^29^ These results indicated that ADFP could promote LAC cell proliferation both in vivo and in vitro. Further, FCM showed that ADFP knock‐down caused G1 phase arrest with a decrease in the proportion of S phase cells. In contrast, ADFP overexpression relieved G1 phase arrest, and the proportion of S phase cells increased. This may be one mechanism by which ADFP regulates LAC cell proliferation.

Then, we explored how ADFP regulates LAC cell proliferation. The p‐Akt/Akt ratio was significantly reduced in ADFP knock‐down LAC cells and increased in ADFP‐overexpressing LAC cells. Consistent with the experiments in vitro, we observed that the p‐Akt/Akt ratio changed in the IHC‐stained tumour tissues of the mouse models described above. We found that the Akt phosphorylation level was related to the ADFP level. We next tested the effects of the Akt phosphorylation inhibitor MK‐2206. The LAC cell proliferation level decreased in the presence of MK‐2206, reaching the same level as observed in ADFP knock‐down LAC cells. Meanwhile, the cell proliferation levels of ADFP‐overexpressing LAC cells increased, which was reversed by MK‐2206. Together, the above results indicated that ADFP promoted LAC cell proliferation by increasing Akt phosphorylation. p‐Akt is the functional form of Akt, and p‐Akt/Akt is often used to evaluate Akt phosphorylation levels.^30^, ^31^, ^32^ Akt hyperphosphorylation can lead to the instability of the genome and even cancer formation.^33^ Studies have shown that PI3K/Akt‐mediated phosphorylation and lipid‐mediated glycogen synthase kinase‐3 (GSK3)/ADFP interactions for GSK3 regulation provided a link between energy homeostasis and cellular functionality.^34^ Therefore, there might be a cross‐talk between ADFP and PI3K/Akt pathways. PI3K activities are modulated by a variety of factors, including lipids. ADFP associated with HIF‐2α promotes lipid storage and endoplasmic reticulum homeostasis under anaerobic conditions.^35^ We consider that ADFP might help tumour cells survive hypoxia by enhancing anaerobic glycolysis.

The present study is the first comprehensive study to establish the functional role of ADFP in LAC cell proliferation and to identify a possible underlying mechanism. Our findings provide new mechanistic insight into the basic theory of LAC progression and identify a potential therapeutic strategy for LAC treatment.

## CONFLICT OF INTEREST

The authors have no conflicts to disclose.

## AUTHOR CONTRIBUTION

SY and XM conceived and designed the project. XM, RS, WW, NZ, SC, BL and SD conducted the experiments and acquired the data. XM, RS and PF analysed the data. XM and WW wrote the paper. All authors approved the final manuscript.

## Supporting information

Fig S1

## Data Availability

Data sharing is not applicable to this article as no new data were created or analysed in this study.

## References

[1] Bray F , Ferlay J , Soerjomataram I , Siegel RL , Torre LA , Jemal A . Global cancer statistics 2018: GLOBOCAN estimates of incidence and mortality worldwide for 36 cancers in 185 countries. CA Cancer J Clin. 2018;68(6):394‐424. 30207593 10.3322/caac.21492

[2] Fidler MM , Bray F , Soerjomataram I . The global cancer burden and human development: A review. Scand J Public Health. 2018;46:27‐36. 10.1177/1403494817715400 28669281

[3] Zhang X , Li W , Hou Y , et al. Comparative membrane proteomic analysis between lung adenocarcinoma and normal tissue by iTRAQ labeling mass spectrometry. Am J Transl Res. 2014;6:267‐280. 24936219 PMC4058308

[4] Zhang XD , Li W , Zhang N , et al. Identification of adipophilin as a potential diagnostic tumor marker for lung adenocarcinoma. Int J Clin Exp Med. 2014;7:1190‐1196. 24955208 PMC4057887

[5] Heid HW , Schnolzer M , Keenan TW . Adipocyte differentiation‐related protein is secreted into milk as a constituent of milk lipid globule membrane. Biochem J. 1996;320(Pt 3):1025‐1030. 9003395 10.1042/bj3201025 PMC1218030

[6] Chong BM , Russell TD , Schaack J , et al. The adipophilin C terminus is a self‐folding membrane‐binding domain that is important for milk lipid secretion. J Biol Chem. 2011;286:23254‐23265. 21383012 10.1074/jbc.M110.217091 PMC3123092

[7] Nakamura N , Fujimoto T . Adipose differentiation‐related protein has two independent domains for targeting to lipid droplets. Biochem Biophys Res Commun. 2003;306:333‐338. 12804567 10.1016/s0006-291x(03)00979-3

[8] Garcia A , Sekowski A , Subramanian V , Brasaemle DL . The central domain is required to target and anchor perilipin A to lipid droplets. J Biol Chem. 2003;278:625‐635. 12407111 10.1074/jbc.M206602200

[9] Miura S , Gan JW , Brzostowski J , et al. Functional conservation for lipid storage droplet association among Perilipin, ADRP, and TIP47 (PAT)‐related proteins in mammals, Drosophila, and Dictyostelium. J Biol Chem. 2002;277:32253‐32257. 12077142 10.1074/jbc.M204410200

[10] Targett‐Adams P , Chambers D , Gledhill S , et al. Live cell analysis and targeting of the lipid droplet‐binding adipocyte differentiation‐related protein. J Biol Chem. 2003;278:15998‐16007. 12591929 10.1074/jbc.M211289200

[11] Orlicky DJ , Degala G , Greenwood C , Bales ES , Russell TD , McManaman JL . Multiple functions encoded by the N‐terminal PAT domain of adipophilin. J Cell Sci. 2008;121:2921‐2929. 18697835 10.1242/jcs.026153 PMC3139108

[12] Hickenbottom SJ , Kimmel AR , Londos C , Hurley JH . Structure of a lipid droplet protein; the PAT family member TIP47. Structure. 2004;12:1199‐1207. 15242596 10.1016/j.str.2004.04.021

[13] Bosma M , Minnaard R , Sparks LM , et al. The lipid droplet coat protein perilipin 5 also localizes to muscle mitochondria. Histochem Cell Biol. 2012;137:205‐216. 22127648 10.1007/s00418-011-0888-x PMC3262136

[14] Listenberger LL , Han X , Lewis SE , et al. Triglyceride accumulation protects against fatty acid‐induced lipotoxicity. Proc Natl Acad Sci U S A. 2003;100:3077‐3082. 12629214 10.1073/pnas.0630588100 PMC152249

[15] Imamura M , Inoguchi T , Ikuyama S , et al. ADRP stimulates lipid accumulation and lipid droplet formation in murine fibroblasts. Am J Physiol Endocrinol Metab. 2002;283:E775‐783. 12217895 10.1152/ajpendo.00040.2002

[16] Ambrosio MR , Piccaluga PP , Ponzoni M , et al. The alteration of lipid metabolism in Burkitt lymphoma identifies a novel marker: adipophilin. PLoS One. 2012;7:e44315. 22952953 10.1371/journal.pone.0044315 PMC3432109

[17] Matsubara J , Honda K , Ono M , et al. Identification of adipophilin as a potential plasma biomarker for colorectal cancer using label‐free quantitative mass spectrometry and protein microarray. Cancer Epidemiol Biomarkers Prev. 2011;20:2195‐2203. 21828233 10.1158/1055-9965.EPI-11-0400

[18] Kurokawa Y , Matoba R , Nakamori S , et al. PCR‐array gene expression profiling of hepatocellular carcinoma. J Exp Clin Cancer Res. 2004;23:135‐141. 15149162

[19] Ostler DA , Prieto VG , Reed JA , Deavers MT , Lazar AJ , Ivan D . Adipophilin expression in sebaceous tumors and other cutaneous lesions with clear cell histology: an immunohistochemical study of 117 cases. Mod Pathol. 2010;23:567‐573. 20118912 10.1038/modpathol.2010.1

[20] Yao M , Huang Y , Shioi K , et al. Expression of adipose differentiation‐related protein: a predictor of cancer‐specific survival in clear cell renal carcinoma. Clin Cancer Res. 2007;13:152‐160. 17200350 10.1158/1078-0432.CCR-06-1877

[21] Morrissey JJ , Mobley J , Figenshau RS , Vetter J , Bhayani S , Kharasch ED . Urine aquaporin 1 and perilipin 2 differentiate renal carcinomas from other imaged renal masses and bladder and prostate cancer. Mayo Clin Proc. 2015;90:35‐42. 25572193 10.1016/j.mayocp.2014.10.005 PMC4317334

[22] Tolkach Y , Luders C , Meller S , Jung K , Stephan C , Kristiansen G . Adipophilin as prognostic biomarker in clear cell renal cell carcinoma. Oncotarget. 2017;8:28672‐28682. 28404922 10.18632/oncotarget.15639 PMC5438682

[23] Cao Q , Ruan H , Wang K , et al. Overexpression of PLIN2 is a prognostic marker and attenuates tumor progression in clear cell renal cell carcinoma. Int J Oncol. 2018;53:137‐147. 29749470 10.3892/ijo.2018.4384 PMC5958875

[24] Hu R , Gupta R , Wang Z , et al. Bioplasmonic paper‐based assay for perilipin‐2 non‐invasively detects renal cancer. Kidney Int. 2019;96:1417‐1421. 31668633 10.1016/j.kint.2019.08.020 PMC7467177

[25] Yao M , Tabuchi H , Nagashima Y , et al. Gene expression analysis of renal carcinoma: adipose differentiation‐related protein as a potential diagnostic and prognostic biomarker for clear‐cell renal carcinoma. J Pathol. 2005;205:377‐387. 15682440 10.1002/path.1693

[26] Takahashi M , Rhodes DR , Furge KA , et al. Gene expression profiling of clear cell renal cell carcinoma: gene identification and prognostic classification. Proc Natl Acad Sci U S A. 2001;98:9754‐9759. 11493696 10.1073/pnas.171209998 PMC55525

[27] Hashimoto Y , Ishida M , Ryota H , et al. Adipophilin expression is an indicator of poor prognosis in patients with pancreatic ductal adenocarcinoma: An immunohistochemical analysis. Pancreatology. 2019;19:443‐448. 30879968 10.1016/j.pan.2019.03.001

[28] Naxerova K , Reiter JG , Brachtel E , et al. Origins of lymphatic and distant metastases in human colorectal cancer. Science. 2017;357:55‐60. 28684519 10.1126/science.aai8515 PMC5536201

[29] Aaltomaa S , Lipponen P , Syrjanen K . Proliferating cell nuclear antigen (PCNA) immunolabeling as a prognostic factor in axillary lymph node negative breast cancer. Anticancer Res. 1993;13:533‐538. 8100128

[30] Jones PF , Jakubowicz T , Pitossi FJ , Maurer F , Hemmings BA . Molecular cloning and identification of a serine/threonine protein kinase of the second‐messenger subfamily. Proc Natl Acad Sci U S A. 1991;88:4171‐4175. 1851997 10.1073/pnas.88.10.4171 PMC51620

[31] Cheng JQ , Godwin AK , Bellacosa A , et al. AKT2, a putative oncogene encoding a member of a subfamily of protein‐serine/threonine kinases, is amplified in human ovarian carcinomas. Proc Natl Acad Sci U S A. 1992;89:9267‐9271. 1409633 10.1073/pnas.89.19.9267 PMC50107

[32] Brodbeck D , Cron P , Hemmings BA . A human protein kinase Bgamma with regulatory phosphorylation sites in the activation loop and in the C‐terminal hydrophobic domain. J Biol Chem. 1999;274:9133‐9136. 10092583 10.1074/jbc.274.14.9133

[33] Brown JS , Banerji U . Maximising the potential of AKT inhibitors as anti‐cancer treatments. Pharmacol Ther. 2017;172:101‐115. 27919797 10.1016/j.pharmthera.2016.12.001 PMC6143165

[34] Liu X , Yao Z . Chronic over‐nutrition and dysregulation of GSK3 in diseases. Nutr Metab (Lond). 2016;13:49. 27493677 10.1186/s12986-016-0108-8 PMC4972972

[35] Qiu B , Ackerman D , Sanchez DJ , et al. HIF2alpha‐dependent lipid storage promotes endoplasmic reticulum homeostasis in clear‐cell renal cell carcinoma. Cancer Discov. 2015;5:652‐667. 25829424 10.1158/2159-8290.CD-14-1507 PMC4456212

