# Supplementary material for: ADFP promotes cell proliferation in lung adenocarcinoma via Akt phosphorylation
Source: J Cell Mol Med. 2020 Nov 29;25(2):827–39. doi: 10.1111/jcmm.16136 (PMC7812254; doi:10.1111/jcmm.16136)
Supplement: Supplementary file 1 — Fig S1 [file JCMM-25-827-s001.docx]

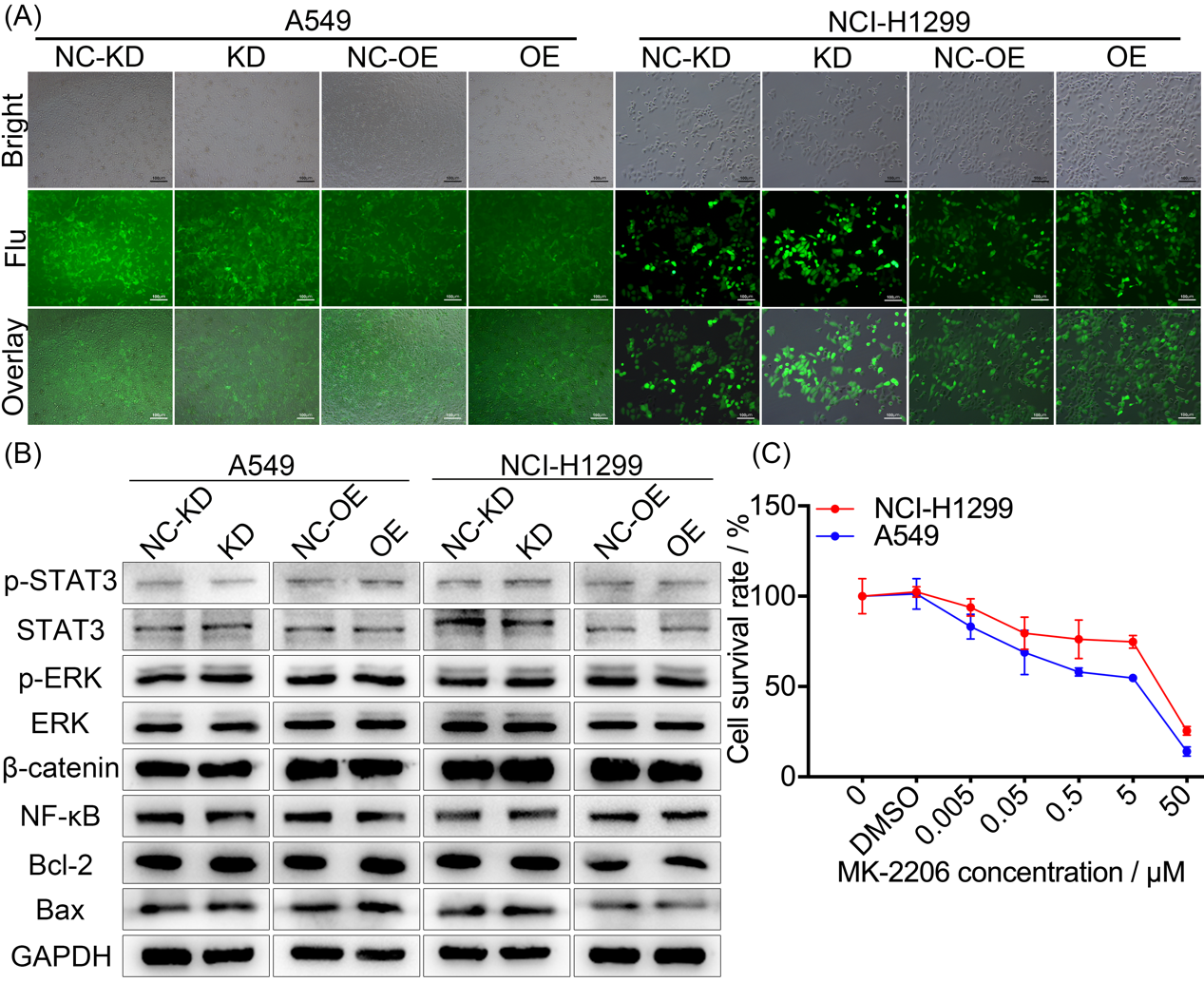


**Figure S1**.

**A**, The transfection efficiencies of lentiviruses into LAC cells were verified by fluorescence microscopy. **B**, Expression levels of p-STAT3, STAT3, p-ERK, ERK, β-catenin, Nf-κB, Bcl-2, and Bax in ADFP‐knockdown and ADFP-overexpressing LAC cells were detected by western blotting. **C**, The effect of MK-2206 at different concentrations on the cell viability of LAC cells was determined by the CCK‐8 assay.
